# Study on Heat Transfer Characteristics and Intelligent Optimization of Protective Clothing Materials in front of the Blast Furnace

**DOI:** 10.1155/2022/5611467

**Published:** 2022-09-26

**Authors:** Zhibin Hu, Yifan Li, Yuanshuai Duan, Yang Han, Aimin Yang, Ziyan Chen, Lijing Wang

**Affiliations:** ^1^College of Science, North China University of Science and Technology, Tangshan 063210, Hebei, China; ^2^The Key Laboratory of Engineering Computing in Tangshan City, North China University of Science and Technology, Tangshan 063210, Hebei, China; ^3^College of Metallurgy and Energy, North China University of Science and Technology, Tangshan 063210, Hebei, China

## Abstract

Special protective clothing should have a certain thermal insulation and protective effect in the production in front of the blast furnace. In order to study the design of special protective clothing for blast furnace foreman, its temperature distribution and internal thickness structure are deeply analyzed. First, based on the existing experimental data, combined with heat transfer and the Fourier law theory, a one-dimensional unsteady heat conduction equation is established. Using the explicit finite difference solution algorithm, the distribution law of the temperature of each fabric layer with time is obtained and the error analysis is performed. The error result is 0.42877%, which shows that the numerical solution is stable and convergent. Then, through the intelligent optimization model of a simulated annealing ant colony (SA-ACO), compared with the single ant colony algorithm (ACO) and a simulated annealing algorithm (SA), it avoids falling into the local optimal solution, reversely solves the optimal thickness of two layers of protective clothing in front of the blast furnace, improves the convergence speed of optimization, and verifies the reliability of the model with a sensitivity test. Finally, this paper actually solves the problem of the protective clothing design and mathematical intelligent model in front of the blast furnace and combines it with numerical simulation software to realize the numerical simulation of temperature distribution and thickness optimization of special protective clothing in front of the blast furnace, so as to provide theoretical guidance for enterprise blast furnace production and development of special protective clothing.

## 1. Introduction

With the development of science and technology in society, the blast furnace smelting process is complex, and the thermal radiation and temperature effects brought about by the blast furnace environment in the production process are also great, such as blast furnace gas combustion explosions, specifically molten metal spattering and leakage, which cause serious damage to the bodies of the staff engaged in this industry [1]. In order to avoid high temperature burns, workers need to wear special protective clothing with good flame retardancy, heat insulation, and polyhydricity, which to some extent is able to achieve the whole protection of the human body in a blast furnace environment [2, 3]. The material of blast furnace preprotective clothing is generally made of aluminum foil, which has good flame retardancy but poor dispersion and resistance to molten metal impact, although it can prevent heat radiation from causing harm to workers.

At present, the design material of protective clothing is mainly aramid fiber, which has good heat and moisture transfer ability and flame retardant fiber effect, and the processing method of multilayer fabric composites is used to further achieve good heat insulation function [4]. Blast furnace protective clothing is generally composed of single-layer or multilayer fabric materials, in the design of protective clothing for blast furnaces, but also to reduce the cost of research and development conditions, the need for numerical simulation of the work clothes under experimental conditions to achieve the best protective effect [5]. A typical protective suit for a blast furnace foreman is composed of four layers of fabric material, the first is in contact with the blast furnace exterior environment and the fourth layer is composed of the gap between the third layer and the dummy skin. The temperature distribution of each layer is obtained by solving a partial differential equation model for the heat transfer process, in which experimental data are available and the temperature distribution of each layer is determined according to the boundary conditions on the model constraints. However, the complexity of solving the heat transfer model for the design of a multilayer blast furnace operating environment special suit is high and there is an urgent need to develop a more rapid and convenient numerical solution algorithm.

In recent years, many researchers and scholars at home and abroad have done research on the material characteristics and thickness optimization of protective clothing for blast furnace foremen. Ghazy et al. established a finite volume model of transient heat transfer in protective clothing consisting of three layers of refractory fabric to study the heat transfer characteristics such as energy content and temperature change of protective clothing but ignored the influence of two gaps between the inner garment and between the garment and skin layers [6]. XU et al. proposed an inverse problem mathematical model of the dynamic heat and humidity transfer thickness of double-layer fabric satisfying human thermal and wet comfort, used the finite difference method to derive numerical solutions of coupled partial differential equations with side value conditions, and proved the uniqueness of their solution existence to reveal the heat transfer law of double-layer fabric [7]. Limei Qi et al. calculated the temperature distribution of three layers of the fabric material based on the law of energy conservation, established a four-parameter exponential model of the skin layer, and cross-validated it [8]. Yuan et al. established the partial differential equations in terms of material temperature, thickness, and working time by an iterative network-based algorithm in the Fourier theory, used finite explicit difference to solve the optimal thickness and time parameters, and considered physical factors such as heat conduction, heat radiation, and heat convection, and the experimental results proved to be more reasonable [9]. Zou et al. proposed an optimization algorithm based on the control variable method and the dichotomous method to obtain the optimal solution for the double-layer thickness of high-temperature protective clothing [10]; Wang et al. proposed the LMNNS method combining back propagation neural network (LMNN) and simulated annealing algorithm (SA) for designing the thickness of each layer of the thermal protective layer [11]. Huang et al. established a heat transfer model for the optimization of the thickness of the protective material and used the optimal thickness of the protective clothing, which was solved by computer numerical simulation using a finite difference discretization method with the required ambient temperature as the constraint [12]. Yu et al. used the heat transfer model and least squares method to solve the optimal thickness of multilayer protective clothing material under high temperature conditions based on the inverse problem [13]. La et al. used the heat transfer model and simulated its numerical solution by finite difference computer, and then used dichotomous method to calculate the optimal thickness of multilayer protective clothing under certain conditions [14]. Li et al. used finite difference numerical simulation of temperature distribution and combined with particle swarm (PSO) optimization algorithm to solve the inverse problem numerically in order to study the inverse problem of solving the thickness of three layers of fabric under the condition of satisfying a certain temperature of human skin to find the optimal thickness of fabric layers [15].

This study has three main innovations and contributions as follows:

1. Based on the existing experimental data, a one-dimensional unsteady heat conduction equation is established through the heat transfer theory. By using the explicit finite difference solution algorithm, the temperature distribution of each fabric layer with time is obtained and the error analysis is performed. The error result is 0.42877%, indicating that the numerical solution is stable and convergent.
2. For the optimization of the fabric thickness of the protective clothing in front of the furnace, we established an intelligent optimization model of simulated annealing ant colony (SA-ACO) coupling. Compared with the single ant colony algorithm (ACO) and simulated annealing algorithm (SA), it avoids falling into the local optimal solution, reversely solves the optimal thickness of two layers of the protective clothing in front of the furnace, improves the convergence speed of the optimization, and verifies the reliability of the model by a sensitivity test.
3. With the existing experimental data, a large number of numerical simulations are carried out by adjusting the algorithm parameters. The experimental results verify the effectiveness and superiority of the simulated annealing ant colony model.

## 2. Heat Transfer Modeling of Protective Clothing Materials for Blast Furnace Foremen

### 2.1. Establishment of an One-Dimensional Heat Conduction Model

According to Fourier's law in heat transfer, the heat *dQ* flowing through an infinitesimal area *dS* along the normal direction *n* within the infinitesimal time *dt* of the furnace worker's protective clothing is positively correlated with the directional derivative *u*_*n*_ of the temperature of the furnace worker's protective clothing material along the normal direction of the surface *dS*, and the obtained fabric protective clothing heat transfer equation is as follows:

(1) $$\begin{array}{c} dQ=-au_{n}dS\;dt\text{,} \end{array}$$

where *Q* represents the heat conducted, *a* is the heat conductivity; the reason for the negative sign is that the energy flows from a high temperature place to a low temperature end, and *S* is the cross-sectional area of the furnace worker's protective clothing.

**Figure 1:**
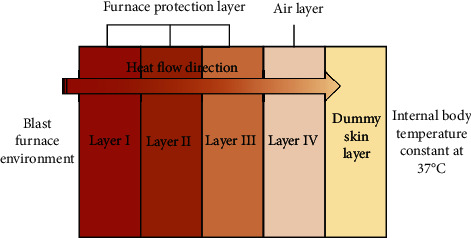
Heat transfer system in each layer of fabric.

In this study, it has been assumed that the protective clothing material object is homogeneous and isotropic, and the heat transfer is perpendicular to the skin layer direction, so the heat transfer system of “blast furnace environment-protective layer-dummy skin layer” can be considered as a one-dimensional problem as shown in Figure 1.

Considering that there is no heat source inside the furnace worker's protective clothing, the heat transfer equation can be derived from (1) in a one-dimensional form [16]:

(2) $$\begin{array}{c} u_{t}=\alpha \Delta u,\Delta u=u_{xx},\alpha =\frac{a}{c\rho }\text{.} \end{array}$$

In equation (2), *α* is the thermal diffusion coefficient, *c* is the specific heat capacity, *a* is the thermal conductivity of the furnace worker's protective clothing, *ρ* is the density, and *u*(*x*, *t*) indicates the temperature at the *x* co-ordinate inside each layer of the fabric material at *t* time.

Since this paper now has to consider isotropic homogeneous materials, which are oriented in the positive direction of the *x*-axis and located at the same temperature in every cross section perpendicular to the *x*-axis, there is no heat exchange between the sides of the dedicated material and the surrounding medium, and there is no heat source inside the furnace worker's protective clothing. In this case, the temperature *u*(*x*, *t*) is a partial differential equation in the coordinates *x*and time *t*, thus giving *u*_*t*_ = *a*/*cρu*_*xx*_.

For the multilayer model, the heat transfer equations for layers I, II, III, and IV can be calculated in this paper as follows:

(3) $$\begin{array}{c} u_{t}=\frac{a_{i}}{c_{i}\rho_{i}}u_{xx},\left(x_{i},t\right)\in \Omega \times \left(0,t_{0}\right),\left(i=1,2,3,4\right)\text{.} \end{array}$$

Then, we consider the starting and boundary conditions of the furnace worker protective clothing material, where the starting conditions are:

(4) $$\begin{array}{c} u\left(t,0\right)=0,u_{i}\left(l_{i},t\right)=u_{i+1}\left(t\right),\left(i=1,2,3,4\right)\text{.} \end{array}$$

The boundary conditions are as follows:

(5) $$\begin{array}{c} u_{1}\left(0,t\right)=u_{0},u\left(x,0\right)=Q\left(t\right)\text{.} \end{array}$$

Based on the above analysis, the differential equation model of heat conduction for each layer of fabric material is established for the abovementioned model as follows:

(6) $$\begin{array}{c} \left\{\begin{array}{c} u_{t}=\frac{a_{i}}{c_{i}\rho_{i}}u_{xx},\left(x_{i},t\right)\in \Omega \times \left(0,t_{0}\right),\left(i=1,2,3,4\right),\\ u_{i}=u_{i+1},x=l_{i},\left(i=1,2,3\right),\\ u\left(t,0\right)=0,u_{i}\left(l_{i},t\right)=u_{i+1}\left(l_{i},t\right),\left(i=1,2,3,4\right),\\ u_{1}\left(0,t\right)=u_{0},u\left(x,0\right)=u_{i}\left(x\right)\text{.} \end{array}\right. \end{array}$$

### 2.2. Finite Difference Method for Solving the One-Dimensional Heat Conduction Model

**Figure 2:**
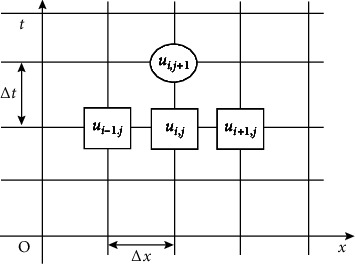
Schematic diagram of spatial and temporal nodes of finite-difference explicit.

In order to solve the heat conduction equation in the previous section, the use of finite differences is considered to solve *u*(*x*, *t*), which is the process of discretizing the difference between the original solution and an equation problem with boundary conditions and then combining it with a computer simulation [17]. The first step is to discretize space and time by dissecting the *xt* plane into a grid, where the *x* and *t* coordinates are divided into *m* and *n* equal parts; here, we have Δ*x*=*h*=*l*/*m*, directional increment *k*=Δ*t*, where *l* is the thickness of the specialized protective clothing fabric material layer, while we let *i* denote the position *x* transverse axis and *j* denote the transverse axis position *t*, with each grid point in the grid corresponding to one of its temperature values.  Figure 2 shows the node diagram of the discrete post-finite difference manifold [18].

Thus, the temperature of the outer side of the dummy skin can be easily known in relation to the different thickness layers and the temperature distribution. In this paper, we use the finite explicit difference approximation to solve the partial differential equation in two-dimensional space.

(7) $$\begin{array}{c} u_{xx}=\frac{u_{i-1,j}-2u_{i.j}+u_{i+1,j}}{\Delta x^{2}}\approx \frac{u_{i-1,j}-2u_{i,j}+u_{i+1,j}}{h^{2}}\text{.} \end{array}$$

Partial differentiation of time yields as follows.

(8) $$\begin{array}{c} u_{t}=\frac{u_{i,j+1}-u_{i,j}}{\Delta t}\approx \frac{u_{i,j+1}-u_{i,j}}{k}\text{.} \end{array}$$

Substituting (7) and (8) into (2) yields as follows:

(9) $$\begin{array}{c} \frac{u_{i,j+1}-u_{i,j}}{k}=\frac{a}{c\rho }\frac{u_{i-1,j}-2u_{i.j}+u_{i+1,j}}{h^{2}}\text{.} \end{array}$$

The terminology is shifted to obtain as follows:

(10) $$\begin{array}{c} u_{i,j+1}=u_{i,j}+\frac{ak}{c\rho h^{2}}\left(u_{i-1,j}+u_{i+1,j}-2u_{i,j}\right)\text{.} \end{array}$$

Let *r*=*ak*/*cρh*^2^ be substituted to obtain the point (*i*, *j*) finite difference approximation formula.

(11) $$\begin{array}{c} u_{i,j+1}=u_{i,j}+r\left(u_{i-1,j}+u_{i+1,j}-2u_{i,j}\right)\text{.} \end{array}$$

And then, we shift the term to obtain as follows:

(12) $$\begin{array}{c} u_{i,j+1}=ru_{i-1,j}+\left(1-2r\right)u_{i,j}+ru_{i+1,j}\text{.} \end{array}$$

In this way, we can find out the outside temperature of the bottom of the I layer, and then, according to the known boundary conditions and initial conditions, we can find out the temperature on the two boundaries *i*=0 and *j*=*n*, and then we can find out the temperature value of each grid point on the coordinates of *j*+1, and then use the initial condition *j*=*t* to calculate the temperature value of each grid point step by step, and then substitute the temperature of the bottom of the next layer based on equation (6), and the solution of the outside temperature of each layer above will cycle through the above steps. However, here, in this paper, we want to ensure that the condition for reaching a stable numerical solution is 0 < *r* ≤ 0.5, so that the value of *u*(*i*, *j*) can be obtained approximately equal to *u*(*x*, *t*).

## 3. Study on the Optimization Model of the Thickness of Protective Clothing for Blast Furnace Foremen

In the algorithmic model for establishing the optimal thickness of the furnace worker's protective clothing, there exists a node of the furnace worker's protective clothing consisting of four layers of fabric material, which changes with the external temperature and the apt ant colony algorithm (ACO) [19]. It is the outer temperature of the dummy skin that is used to find the optimal thickness of layer II and IV nodes by searching for pheromone updates, but since the solution generated by each ant is randomly generated by probability, this may make the pheromone update also randomly and not necessarily get the optimal thickness, thus falling into a local optimal solution. Based on this, the SA [20] algorithm is proposed in this paper to optimize the range of ant search solutions in the ACO algorithm, which is an inspirational algorithm [21–23], and its principle originates from the thermodynamic problem of heating a solid to a certain temperature and then slowly cooling it down to a stable state, i.e., the more ordered solid crystal goes through the process of a disordered liquid state and then to a more ordered state. The interval probability of acceptance of the optimal solution can be obtained using the Metropolis sampling process to update the pheromone. This translates into the simulated annealing-ant colony algorithm (SA-ACO) [24] problem of finding the optimal solution in the solution space.

(1) Transfer probability of ants: The transfer probability of layer *i* and layer *j* in the ACO algorithm is calculated as follows:
  (13) $$\begin{array}{c} P_{ij}^{k}=\left\{\begin{array}{c} \frac{m_{ij}{\left(t\right)}^{\alpha }\cdot n_{ij}{\left(t\right)}^{\beta }}{\sum m_{ij}{\left(t\right)}^{\alpha }\cdot n_{ij}{\left(t\right)}^{\beta }},j\in a_{k}\\ 0,\;j\notin a_{k} \end{array}\right.,\left(i,j=1,2,3,4\right)\text{.} \end{array}$$
  The formula (13) *m*_*ij*_ is the pheromone strength of the *i* layer to the *j* layer, and *n*_*ij*_ is the *i* layer to the *j* layer visibility intensity, *P*_*ij*_^*k*^ for ant *k* from the transfer probability of the *i* layer and the *j* layer, *α* is a positive inspiration factor, *β* is the desired inspiration factor, *a*_*k*_ is the *k* knows the ant current fabric layer, where *m*_*ij*_(0) = *m*_0_ is the initial pheromone, and the larger *m*_*ij*_(*t*)^*α*^*·n*_*ij*_(*t*)^*β*^ is, the shorter the distance between layer *i* and layer *j*, the higher the pheromone intensity. After *n* calculations, each time a layer to be visited is determined, so that a path is formed. A population with *m* ants, then it is possible to obtain *m* feasible solutions [25]. If the ant at the *i* layer is located close to the location with the highest pheromone content in the current population, then the improved ACO algorithm is performed to transfer the probability formula as follows:
  (14) $$\begin{array}{c} P_{i}=1-\frac{m_{i}\left(t\right)}{\max_{\lambda \in \left[1,j\right]}m_{s}\left(t\right)},\left(i=1,2,3,4\right)\text{,} \end{array}$$
  where *m*_*i*_(0) = *G*(*x*), where *G*(*x*) is the objective function to be searched for the optimum. From the transfer probability formula (14), it can be seen that the closer the pheromone of the ant's current position is to the optimal value, the smaller *p*_*i*_ is, and on the contrary, it should tend to search in a wide range.
  (15) $$\begin{array}{c} x\left(t+1\right)=\left\{\begin{array}{c} x\left(t\right)+r\lambda ,p_{i}<p_{0,}\\ x\left(t\right)+\frac{r\bar{x}}{2},p_{i}\geq p_{0}\text{,} \end{array}\right. \end{array}$$
  where *r* ∈ [−1,1] is a random number, *p*_0_ is a transfer probability constant, *λ* = 1/*t*; when *p*_*i*_ < *p*_0_, *λ* decreases with the number of iterations and is a local search; on the contrary, *p_i_* ≥ *p*_0_ is a global search.
(2) Pheromone updates [26]: After performing Metropolis sampling, its ant colony algorithm updates the way
  (16) $$\begin{array}{c} \left\{\begin{array}{c} m_{ij}\left(t+1\right)=\left(1-\xi \right)m_{ij}\left(t\right)+\Delta m_{ij}\\ \Delta m_{ij0}=\overset{m}{\underset{k=1}{\sum }}\Delta m_{ij}^{k} \end{array}\right.,0<\xi <1\text{.} \end{array}$$
  In this paper, this idea can be used to adjust the pheromone at the location of ant *i*. The improved ant colony algorithm is calculated as follows:
  (17) $$\begin{array}{c} m_{i}\left(t+1\right)=\left(1-\xi \right)m_{i}\left(t\right)+\omega G\left(x\right)\text{,} \end{array}$$
  where *ω* here is a constant that represents the pheromone released by the ant in one cycle. The pheromone of the current position of the ant and the formula for the next iteration can be obtained to construct an ant colony algorithm model to solve the problem of determining the optimal thickness of the furnace worker's protective clothing.
(3) Metropolis guidelines: the core of the simulated annealing algorithm (SA) is in the Metropolis criterion [27], which is

(18) $$\begin{array}{c} P\left(\Delta f\right)=\left\{\begin{array}{c} 1,\Delta f<0\text{,}\\ e^{-\frac{\Delta f}{KT}},\Delta f\geq 0\text{.} \end{array}\right. \end{array}$$

In equation (18), *K* is the Boltzmann constant, *T* is the current absolute temperature, and *P*(Δ*f*) is the probability of accepting the new state solution. At this point, there exists a random number *γ* ∈ [0,1], if *P*(Δ*f*) > *γ*, the new state arrangement is accepted; if *P*(Δ*f*) ≤ *γ*, the original state arrangement is retained.

**Figure 3:**
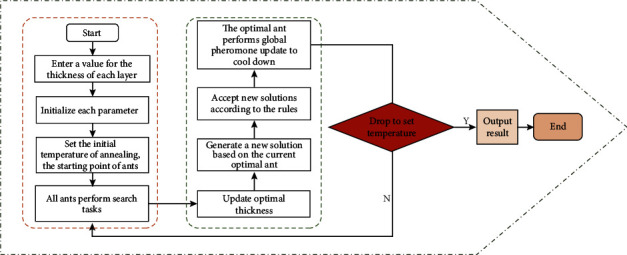
SA-ACO algorithm flowchart.

This completes the combination of the SA-ACO algorithm, and the flowchart of its solution algorithm is shown in Figure 3.

## 4. Analysis of Results

### 4.1. Simulation Results and Analysis of Heat Transfer Characteristics of Protective Clothing Materials for Blast Furnace Foremen

Based on the heat transfer model study of the blast furnace foreman's protective clothing material, it is assumed that thermal convection, thermal radiation, and thermal dissolution are not considered and the temperature change of each fabric layer of the furnace worker's protective clothing is continuously varied. According to the density *ρ*, specific heat capacity *c*, and heat transfer coefficient *a* given by the material of each layer of the furnace worker's special suit, the values taken on different materials are different, respectively *ρ*_*i*_, *c*_*i*_, *a*_*i*_(*i*=1,2,3,4), and the values of the main parameters of the heat transfer characteristics of the material of the blast furnace foreman's protective suit here are shown in Table 1.

Then, the established one-dimensional unsteady heat conduction model is solved by combining the bounded difference to obtain an approximate numerical solution and then dropping the substitution again. We put the initial conditions of formula (6) to take the Fourier sinusoidal transformation to translate the initial value problem of the normalized equation containing the parameter [28, 29].

In the finite difference equation,

(19) $$\begin{array}{c} \frac{d\int_{0}^{+\infty }u\left(x,t\right)\sin\;\omega x\;dx}{dt}=\frac{a}{c\rho }\left[\omega -\omega^{2}\int_{0}^{+\infty }u\left(x,t\right)\sin\;\omega x\;dx\right]\text{.} \end{array}$$

The solution is as follows:

(20) $$\begin{array}{c} U\left(\omega ,t\right)=e^{-\frac{at\omega^{2}}{c\rho }}\int_{0}^{t}\frac{a\omega }{c\rho }e^{ay\omega^{2}/cp}dy\text{.} \end{array}$$

Then, taking the Fourier inverse transform of (20), its solution can be obtained using the following formula:

(21) $$\begin{array}{c} u\left(x,t\right)=\frac{x}{2\surd a\pi /c\rho }\int_{0}^{t}\frac{1}{\sqrt{{\left(t-y\right)}^{3}}}e^{-\frac{c\rho x^{2}}{4a\left(t-y\right)}}dy\text{.} \end{array}$$

**Figure 4:**
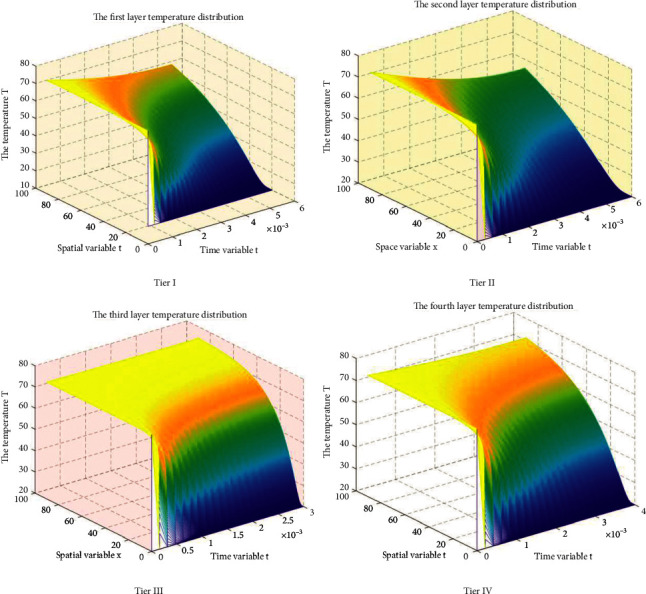
Temperature distribution inside the protective clothing of the furnace workers in layers I-IV.

The temperature distribution of each layer was solved by MATLAB simulation. At an ambient temperature of 75°C and a working time of 90 minutes, the differences in thermal conductivity and specific heat capacity of the different layers of the protective clothing for furnace workers resulted in significant differences in the temperature of the embodied dummy's outer skin. See Figure 4 for a two-dimensional plot of temperature distribution versus temperature versus time for each layer.

Figure 4 shows the temperature change of each subinterface furnace worker's protective material. The temperature at each node has a rapid growth trend but all to a node after the stabilization. When layer I comes in contact with the external environment with the passage of time and space location, the temperature on the outside of the dummy skin obviously shows a trend of first decreasing and then increasing, because it is in direct contact with the external environment; at the same time, the contact between layer IV and the skin shows the opposite trend; at the same time, with the increase of the coordinate position, the temperature at the node rises gradually decreases; for the same node, the temperature at the node gradually increases, but the rate of temperature change gradually decreases, and eventually all the node temperatures converge to a stable temperature value.

**Figure 5:**
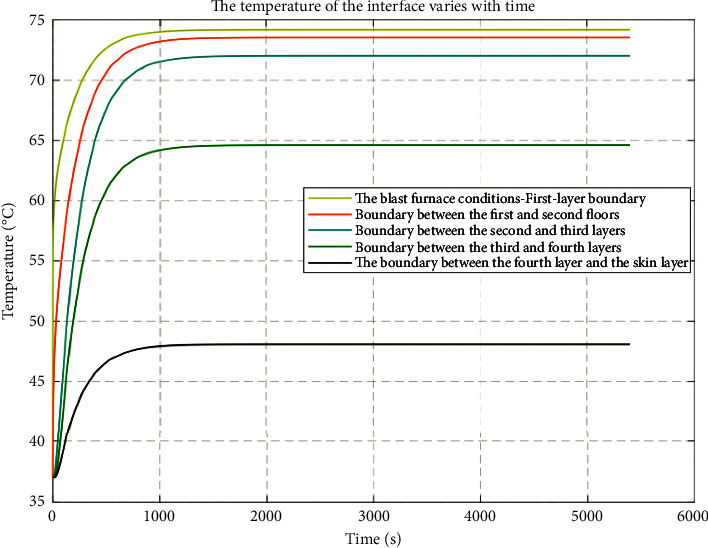
Changes in the temperature of the protective clothing material of the boundary furnace.

Figure 5 shows the two-dimensional image of temperature change with time, which can be found to blast furnace environment-I–II–III layer of each boundary rising trend more obvious, while the III-IV layer boundary began to rise slowly and slowly tend to stabilize, the IV layer-skin layer boundary temperature slowly rising is the slowest.

Through finite differences, it is only necessary to find an approximate solution but it is important to the last result, thereby performing error analysis.

**Figure 6:**
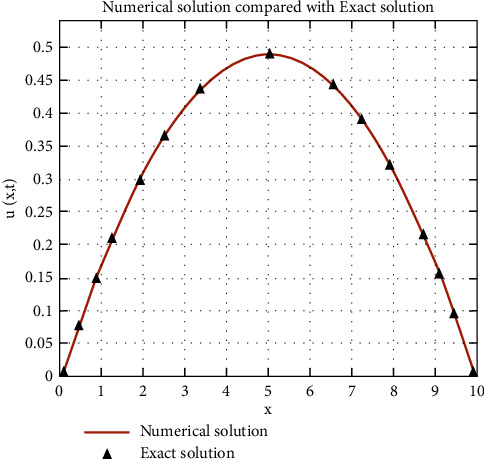
Comparison of numerical and exact solutions.

Figure 6 shows that the result of running through the MATLAB program is that the maximum relative error of numerical solution and precision is very small, at 0.42877%, and the resulting result of the numerical solution is stable convergence. Therefore, the temperature distribution change obtained herein is reasonable and feasible.

After obtaining the temperature distribution law of each layer of the fabric, in order to further study the heat transfer characteristics of the blast furnace, this article also needs to design the dummy in a particular environment, so that it can better serve production and development of precursor protective clothing in blast furnace. To design safer protective clothing for blast furnace foremen, it is necessary to find the optimum thickness of the protective clothing. Based on a one-dimensional conduction model and the temperature distribution law of the furnace foremen's protective clothing, the effect of temperature and time variation on the thickness of the furnace foremen's protective clothing is obtained.

### 4.2. Results and Analysis of Thickness Optimization of Protective Clothing for Blast Furnace Prefurnace Workers

In order to determine the effect of the main factors in the simulated annealing-ant colony algorithm (SA-ACO) on the outer skin temperature of the dummy, first, in this paper, a global sensitivity analysis method is to be applied to the model, and then a sensitivity analysis is to be done by fitting a function-based on a heat transfer model with ambient temperature, layer II and IV thicknesses, and working time as input variables and outer skin temperature as an output variable [30]. By calling the existing library of MATLAB, we make the curve graph and observe whether the points of the curve are consistent.

**Figure 7:**
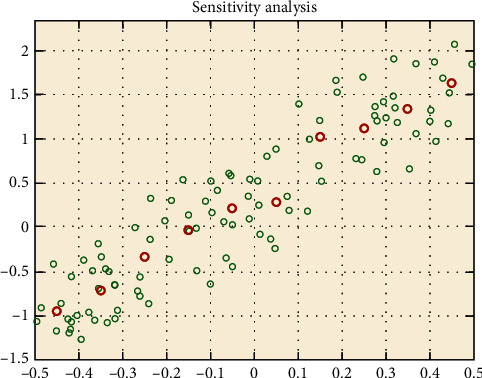
Sensitivity test.

Figure 7 shows that the fitting relationship to the curves is basically consistent, and the analysis shows that the changes in ambient temperature, II and IV thicknesses, and working time do not affect the outer skin temperature, indicating that the algorithm model passes the sensitivity test. In turn, the thickness search is performed to find the optimal thickness to ensure the best outer skin temperature and material properties of the furnace worker's protective clothing.

**Figure 8:**
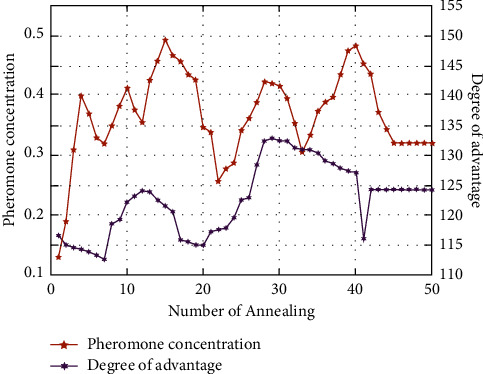
SA-ACO algorithm total dominance curve and optimal path pheromone concentration.

Based on the hybrid optimization of the SA-ACO algorithm, the trends of annealing number and dominance with pheromone concentration can be obtained, as shown in Figure 8. It can be obtained that when the SA-ACO algorithm reaches the 28th annealing, then the dominance value has exceeded the dominance at convergence, and the pheromone concentration of the path is relatively small at this time, and at the 14th annealing, the pheromone concentration of the algorithm exceeds the pheromone concentration of the path at convergence but the dominance is not large at this time. Therefore, a combination of dominance and pheromone concentration is considered in path selection, not just a single one, so that local optimal solutions can be avoided.

**Figure 9:**
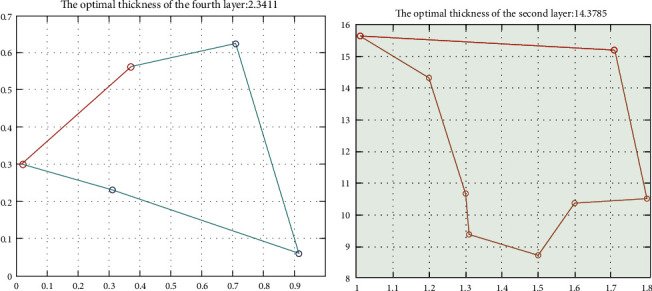
II and IV optimal thickness of simulated annealing-ant colony algorithm.

The dummy was placed in a blast furnace environment with a laboratory temperature of 80°C and a working time of 30 minutes to carry out the experiment, and it was required that the outer skin temperature of the middle dummy did not exceed 47°C, and the time exceeding 44°C did not exceed 5 minutes. The optimal thicknesses of layer II and layer IV were obtained for their given objective functions by optimally solving with the SA-ACO algorithm as shown in Figure 9.

The optimal thickness of layer IV is 2.3411 mm and the optimal thickness of layer II is 14.3785 mm. Finally, the training results of the SA-ACO coupling algorithm are tested with the existing experimental data and compared with the results of the conventional SA and ACO algorithms, and the comparison results are shown in Table 2. The comparison found that the thickness-seeking search accuracies of layer II and layer IV of the coupled SA-ACO algorithm were 92.12% and 90.15%, respectively, which were higher than the accuracies of 89.23% and 86.64% of the traditional SA algorithm and 90.14% and 88.21% of the ACO algorithm.

**Figure 10:**
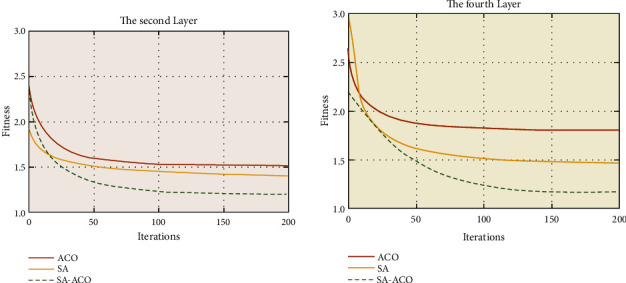
Curves of the target iterative process of the three algorithms.

Figure 10 gives the results of the comparison of the fitness values of the three algorithms with the number of iterations for the thickness finding of the furnace worker's protective clothing for the existing experimental data. The horizontal coordinate represents the number of iterations and the adaptation degree decreases as the number of iterations increases. When the number of iterations exceeds 50, the loss values of the three algorithms tend to stabilize but it can be seen from Figure 10 that the adaptation degree of the SA-ACO coupled algorithm is significantly lower than that of the traditional SA algorithm and the ACO algorithm, and its decreasing speed is also higher than that of the single SA and ACO algorithms.

There are three algorithms in the process of finding the optimum for layer II and layer IV thicknesses based on the available experimental data. Figure 10 shows that the ACO algorithm falls into a local optimum for fewer and more iterations but the SA-ACO algorithm makes the outer skin temperature suitable and globally optimal for the right number of iterations. This shows that the SA-ACO coupling algorithm improves the search accuracy of the traditional ACO and SA algorithms while also correspondingly improving the calculation speed of the model, and the SA-ACO coupling algorithm is applied to the problem of furnace worker clothing design with good results and satisfactory accuracy, which has a certain theoretical guidance significance for the production of special protective clothing for furnace workers in the enterprise blast furnace.

## 5. Conclusions

1. A one-dimensional nonstationary heat transfer model is established, and the temperature distribution of each fabric layer and air layer of the blast furnace worker's protective clothing is solved by combining the finite difference method, and the reliability of the results is verified by error analysis.
2. The modal SA-ACO algorithm is used to improve the local optimum solution that is trapped in the process of finding the optimum, and the sensitivity test is done to verify the reliability of the model to achieve the thickness finding of the blast furnace foreman's protective clothing layer II and layer IV.
3. On academic value, this paper solves the key problems of designing and mathematical intelligent modeling of blast furnace foreman protective clothing, which can be further combined with numerical simulation software to realize numerical simulation of temperature distribution and thickness finding for blast furnace foreman protective clothing, providing a theoretical guidance basis for enterprise blast furnace production and development of blast furnace foreman protective clothing.

## Figures and Tables

**Table 1:** Operator protective equipment material heat transfer characteristics input parameter value.

Layering	Density (kg/m^3^)	Brc (J/kg.^0^ C)	Thermal conductivity (W/m.^0^ C)	Thickness (mm)
I layer	300	1377	0.082	0.6
II layer	862	2100	0.082	0.6–25
III layer	74.2	1726	0.045	3.6
IV layer	1.18	1005	0.028	0.6–6.4

**Table 2:** Thickness measurement accuracy of three algorithms for furnace worker protective clothing.

Algorithm	Layer II thickness search accuracy (%)	Layer IV thickness search accuracy (%)
SA	89.23	86.64
ACO	90.14	88.21
SA-ACO	92.12	90.15

## Data Availability

The data used to support the findings of this study are available from the author upon request.
